# Targeting Mitochondrial Integrity as a New Senolytic Strategy

**DOI:** 10.14336/AD.2024.1100

**Published:** 2025-12-18

**Authors:** Eliska Vacurova, Edita Vlachova, Jan Stursa, Klara Bohacova, Tereza Havrlantova, Vojtech Skop, Barbora Judita Kasperova, Lukas Werner, Jiri Neuzil, Martin Haluzik, Sona Stemberkova Hubackova

**Affiliations:** ^1^Institute of Biotechnology, Czech Academy of Sciences, Prague-West, Czech Republic.; ^2^Diabetes Centre, Institute for Clinical and Experimental Medicine, 140 21 Prague, Czech Republic.; ^3^Faculty of Science, Charles University in Prague, 121 08 Prague, Czech Republic.; ^4^Centre for Experimental Medicine, Institute for Clinical and Experimental Medicine, 140 21 Prague, Czech Republic.; ^5^Department of Biochemistry and Microbiology, University of Chemistry and Technology, Prague, Czech Republic.; ^6^First Faculty of Medicine, Charles University in Prague, 121 08 Prague, Czech Republic.; ^7^School of Pharmacy and Medical Science, Griffith University, Southport, Qld, Australia.

**Keywords:** Mitochondria, senescence, senolytics, ferroptosis, mitochondrial integrity

## Abstract

Aging, characterized by accumulation of senescent cells, is a driving factor of various age-related diseases. These conditions pose significant health risks globally due to their increasing prevalence and serious complications. Reduction of senescent cells therefore represents a promising strategy promoting healthy aging. Here we demonstrate that targeting tamoxifen to mitochondria via triphenyl and tricyclohexyl phosphine selectively eliminates senescent cells. Our findings show a complex effect of mitochondrially targeted tamoxifen on mitochondrial function and integrity of senescent cells, including inhibition of oxidative phosphorylation and activity of respiratory complex IV. These changes result in activation of ferroptosis as the major mode of cell death, which results in rejuvenation of tissues. Targeting mitochondria of senescent cells represents a general senolytic strategy and may extend the healthspan and improve the quality of life in aging populations.

## INTRODUCTION

**Figure 1.**
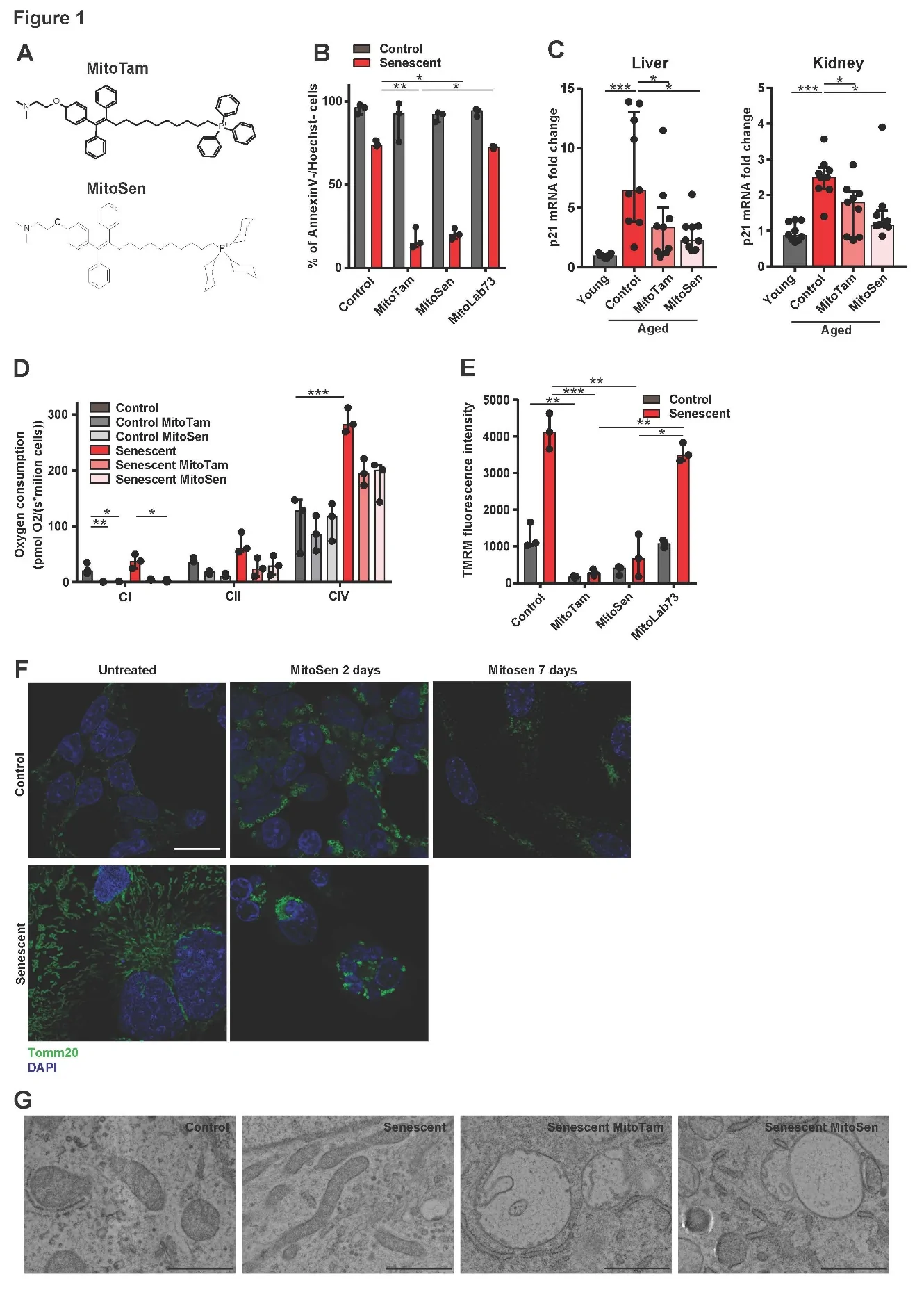
**Mitochondrially targeted senolytic agents affects mitochondrial function in senescent cells**. (**A**) Molecular structure of MitoTam and MitoSen. (**B**) RPE-1 control and senescent cells (induced by 100 μM BrdU every 48h for 8 days) were treated with MitoTam, MitoSen or MitoLab73 (1μM for all compounds) for 48h. The percentage of viable cells was determined by annexinV/Hoechst staining followed by FACS analysis. (**C**) 18-month-old Balb-c mice (Aged, n=9) were treated i.p. once per week for a period of 4 weeks with MitoTam or MitoSen (2 mg/kg of body weight) dissolved in 4 % EtOH in corn oil, or with the vehicle (Control). 2-month-old Balb-c mice (Young, n=9) were used as a control. Liver and kidney were excised and assessed for the expression of *p21^cip1/waf1^* mRNA by RT-qPCR. (**D**) RPE-1 control, and senescent cells (induced as in B) were treated with MitoTam or MitoSen (1 μM) for 24h. CI and CII-dependent respiration, and CIV activity were analyzed by Oxygraph 2K. (**E**) Mitochondrial membrane potential was analyzed by potentiometric fluorescent dye tetramethylrhodamine methyl ester (TMRM; 50 nM, 15 min) and analyzed by FACS. (**F**) Mitochondrial morphology in control and senescent RPE-1 cells treated with MitoSen (1 μM) for 24 h and 7 days, respectively, was documented by Tomm20 immunofluorescent staining, with DAPI denoting cell nuclei. The scale bar indicates 10 μm. (**G**) Mitochondrial morphology in control and senescent RPE-1 cells treated with MitoTam or MitoSen (1 μM) for 24h was documented by electron microscope. The scale bar indicates 1μm. Data in B, D, E and G are presented as median values with interquartile range from three independent experiments. Data in C are represented as median values with interquartile range.

Cellular senescence and aging are major contributors to the development of age-related diseases, which are becoming increasingly prevalent worldwide as life expectancy rises. Senescent cells, accumulating in tissues during aging, are characterized by a cessation of cell division and the secretion of inflammatory factors, known as the senescence-associated secretory phenotype (SASP). This SASP leads to chronic inflammation, tissue dysfunction, and disruption of normal cellular environment, creating a fertile ground for various age-related diseases such as cardiovascular diseases, type 2 diabetes, cardio-renal diseases, and neurodegenerative disorders like Alzheimer’s disease [1]. These conditions are not only leading causes of mortality and morbidity but also impose significant economic and healthcare burdens globally. Understanding and targeting cellular senescence is therefore crucial for developing strategies to prevent or mitigate age-related diseases, thereby improving quality of life and reducing healthcare costs worldwide. Several studies and clinical trials have suggested beneficial effects of senolytic agents [2]. However, current treatments target specific proteins or pathways that may not be present in all senescent cells, potentially leading to disease recurrence after therapy. Given that altered mitochondrial function contributes significantly to the aging process and senescent phenotype, targeting mitochondria could effectively eliminate these cells in senescence-related diseases. In our recent research, we discovered that mitochondrially targeted tamoxifen (MitoTam), i.e., tamoxifen tagged with the mitochondrial vector triphenylphosphonium (TPP) (Fig. 1A), specifically targets senescent cells [3] to reduce obesity and reverse metabolic imbalance [4]. However, the precise cellular mechanism through which MitoTam specifically eliminates senescent cells has not been clarified.

## MATERIALS AND METHODS

### Material

Q-VD-Oph (S7311), Z-VAD-FMK (S7023) and Necrostatin-1 (S8037) were purchased from Selleckchem (Cologne, Germany). 3-Methyladenine (M9281), Ferrostatin-1 (SML0583) and RSL3 (SML2234) were purchased from Sigma (Rahway, NJ, USA).

### Cell lines

Human primary retinal pigment epithelial cells (RPE-1) and human umbilical vein cells (EA.hy962) were sourced from ATCC (Manassas, VA, USA). Cells were maintained in the DMEM medium supplemented with 10% foetal bovine serum (ThermoFisher Scientific, Waltham, MA, USA), 4.5 g/L glucose (Sigma, Rahway, NJ, USA) and 100 U/mL penicillin and 100 μg/mL streptomycin sulfate (Sigma, Rahway, NJ, USA). The cells were kept at 37 °C under 5 % CO_2_ in a humidified atmosphere.

### Assessment of cell viability

The medium containing dead cells was collected into clear tube, adherent cells were trypsinized, re-suspended in the medium containing dead cells and centrifuged at 1,000 x g for 3 min. The pellet was resuspended in 200 μL of annexin V binding buffer containing 0.3 µL of annexin V-Dyomics 647 (Apronex, Vestec, Czech Republic), and incubated for 20 min at 4 °C. Hoechst 33258 (5 μg/mL) was added before the cells were analyzed by flow cytometry (BD LSR Fortessa, San Jose, CA, USA). Cell viability was expressed as the percent of the annexin V-negative/Hoechst-negative fraction. Alternatively, WST-1 assay for cell proliferation and viability (Sigma, Rahway, NJ, USA) was used. For this analysis, culture medium without phenol red was used prior adding detection reagent. Cells were incubated for 2 h. Signal was detected using the BioTek Epoch Microplate Spectrophotometer (Agilent, Santa Clara, CA, USA).

### Evaluation of mitochondrial membrane potential

To assess mitochondrial membrane potential, cells were treated with tetramethylrhodamine methyl ester (TMRM; 50 nM) for 15 min prior to analysis by flow cytometry. Carbonyl cyanide *m*-chlorophenyl hydrazine (CCCP; 20 μM) was added 5 min before TMRM to see specific suppression of mitochondrial membrane potential. Cells without added TMRM probe were used as a negative control.

### Evaluation of respiration

The high-resolution Oxygraph-2k respirometer (Oroboros Instruments, Innsbruck, Austria) was used to assess routine respiration. Cells were trypsinized, washed with PBS, re-suspended at 2x10^6^ cells per mL in the Mir05 medium (0.5 mM EGTA, 3 mM MgCl_2_, 60 mM K-lactobionate, 20 mM taurine, 10 mM KH_2_PO_4_, 110 mM sucrose, 1 g/L essentially fatty acid-free bovine serum albumin, 20 mM Hepes, pH 7.1 at 30 °C) and transferred to the chamber of the Oxygraph-2k instrument. Respiration evaluation was performed at 37 °C as follows. After signal stabilization, the chamber was closed and the oxygen consumption started to rise showing the level of basal respiration. To measure complex I (CI) and complex II (CII) dependent respiration, malonate (10 µg/ml) and rotenone (2 µg/ml) were added to inhibit CII and CI, respectively. Level of CI and CII respiration after the addition of glutamate/malate (5/5 µg/ml) and succinate c (10 µg/ml), respectively, was determined. After OXPHOS inhibition by antimycin A (2 µg/ml) the activity of complex IV was measured by tetramethyl-*p*-phenylene diamine (0.5mM) in the presence of ascorbate (2mM). Data were evaluated using DatLab5 software (Oroboros Instruments, Innsbruck, Austria).

### Electrophoresis and western blotting

Cells were washed twice with PBS, harvested into Laemmli SDS sample lysis buffer (2% SDS, 50 mM Tris-Cl, 10% glycerol in double distilled H_2_O) and sonicated (2x10 s at 1 micron amplitude with 10 s cooling interval) using the Soniprep 150 instrument (MSE, London, UK). Protein concentration was estimated using the BCA method (Pierce Biotechnology). Cell lysates were supplemented with 100 mM DTT (Sigma, Rahway, NJ, USA) and 0.01% bromophenol blue (Sigma, Rahway, NJ, USA) before separation by SDS-PAGE. The same amount of protein (50-70 μg) was loaded into each well. The protein was transferred onto a nitrocellulose membrane using wet transfer and detected by specific antibodies combined with horseradish peroxidase-conjugated secondary antibodies (goat anti-rabbit or goat anti-mouse). Peroxidase activity was detected using the ECL Western Blotting Substrate or the SuperSignal West Femto Extended Duration Substrate (ThermoFisher Scientific, Waltham, MA, USA). anti-KEAP1 (#8047), anti-FH1 (#4393), anti-SLC7A11 (#12691) and anti-GTX4 (#52455) were purchased from Cell Signaling (Danvers, MA, USA), anti-MT-CO1 (ab14705), anti-MT-CO2 (ab110258), anti-MT-CO3 (ab110259) and anti-LPCAT3 (ab232958) were purchased from Abcam (Cambridge, UK), anti-NDUFV1 (PA5-67373) was purchased from ThermoFisher Scientific (Waltham, MA, USA) and anti-PLCB4 (HPA00795) from Sigma (Rahway, NJ, USA). All antibodies were diluted 1:1,000 in 2.5% non-fat milk. HPR conjugated β-actin (PA1-183-HPR; ThermoFisher Scientific, Waltham, MA, USA) was used as a loading control. IgG-HRP anti-rabbit (170-6515) and anti-mouse (170-6516) secondary antibodies produced in goat were purchased from BioRad Laboratories (Hercules, CA, USA). Secondary antibodies were diluted 1:10,000 in 2.5% non-fat milk.

### Indirect immunofluorescence

Cells grown on glass coverslips were fixed with 4 % paraformaldehyde (PFA; VWR, Radnor, PA, USA) and permeabilized with 0.1 % Triton X-100 (Sigma) in two consecutive steps, each at room temperature (RT) for 15 min. After washing with PBS, cells were incubated in 10 % FBS (diluted in PBS) for 30 min to block unspecific signals. Cells were incubated for 1 h at RT with diluted primary antibodies anti-TOMM20 (EPR15581-54, Abcam) or 53BP1 (MAB3802, Sigma), washed extensively with PBS/0.1 % Tween 20, and incubated for 1 h at RT with the secondary goat anti-rabbit Alexa Fluor 488 IgG (A11006, ThermoFisher). Coverslips were mounted in Mowiol containing 4',6-diamidino-2-phenylindole (DAPI) to stain nuclei, and the signal was detected using the Leica SP8 FLIM confocal microscope (Leica Microsystems, Wetzlar, Germany).

### Quantitative real time PCR (RT-qPCR)

Small pieces of tissue (1-2 mm^3^) were placed into 500 μL of RNAzol (BioRad), homogenized (3 x 40 s at 5,600 rpm) using the Precellys 24 homogenizator (Bertin Instruments, Montigny-le-Bretonneux, France) and cleared by centrifugation; total RNA was isolated according to the manufacturer´s protocol. First strand cDNA was synthesized from 1 μg of total RNA with random hexamer primers using Revert Aid First strand cDNA Synthesis Kit (ThermoFisher Scientific, Waltham, MA, USA). RT-qPCR was performed using the CFX384 Touch Real-Time PCR Detection System (BioRad) with 5xHOT FIREPol Evagreen qPCR Supermix GreenE dye (Solis Biodyne, Tartu, Estonia). The relative quantity of cDNA was estimated by the ΔΔCT method, data were normalized to β-actin. The following primers were purchased from Sigma: mouse p21: forward 5´- TCG CTG TCT TGC ACT CTG GTG T-3´, reverse 5´- CCA ATC TGC GCT TGG AGT GAT AG-3´; mouse p16: forward 5´- CGG TCG TAC CCC GAT TCA G-3´, reverse 5´- GCA CCG TAG TTG AGC AGA AGA G-3´; mouse β-actin: forward 5´- CAT TGC TGA CAG GAT GCA GAA GG-3´, reverse 5´-TGC TGG AAG GTG GAC AGT GAG G-3´.

### Transmission electron microscopy (TEM)

TEM was performed according to standard protocol. In brief, cells were grown on cover-slips, fixed with 2.5% glutaraldehyde (Sigma) overnight, and post-fixed with 1% OsO4 (Sigma) made up in Sorensen’s phosphate buffer (0.1 M, pH 7.2-7.4), dehydrated in acetone series, and embedded in Epon-Durcupan (Sigma). Ultrathin sections (~70-90 nm) were cut, contrasted with uranyl acetate (Ladd Research Industries, Williston, VT, USA), and examined in the JEM-1400 FLASH transmission electron microscope (Jeol) at 80 kV. Images were captured with 2kx2k FLASH CMOS camera.

### Proteomic analysis

Cell pellets were lysed in 100 mM TEAB containing 2% SDC and boiled for 5 min. Protein concentration was determined using the BCA protein assay kit (ThermoFisher Scientific), and 20 µg of protein per sample was used for MS sample preparation. Cysteine residues were reduced with 5 mM (final concentration) of TCEP (60 °C, 60 min) and blocked with 10 mM final concentration of MMTS (10 min at RT). Samples were digested with trypsin (trypsin/protein ratio 1/30) at 37 °C overnight. Samples were then acidified with TFA (1% final concentration). SDC was removed by extraction to ethylacetate, and peptides were desalted using in-house made stage tips packed with C18 disks (Empore, Helsinki, Finland) as described.[5]

Nano reversed-phase columns (EASY-Spray column, 50 cm x 75 µm ID, PepMap C18, 2 µm particles, 100 Å pore size) were used for LC/MS analysis. Mobile phase buffer A was composed of water and 0.1% formic acid. Mobile phase B was composed of acetonitrile and 0.1% formic acid. Samples were loaded onto the trap column (C18 PepMap100, 5 μm particle size, 300 μm x 5 mm, ThermoFisher Scientific) for 4 min at 18 μL/min, with loading buffer composed of water, 2% acetonitrile and 0.1% trifluoroacetic acid. Peptides were eluted with mobile phase B gradient from 4% to 35% B in 120 min. Eluting peptide cations were converted to gas-phase ions by electrospray ionization and analyzed on a Thermo Orbitrap Fusion (Q-OT- qIT, ThermoFisher Scientific). Survey scans of peptide precursors from *m/z* 350 to 1400 were performed in the Orbitrap at 120K resolution (at *m/z* 200) with a 5x10^5^ ion count target. Tandem MS was performed by isolation at 1.5 Th with the quadrupole, HCD fragmentation with normalized collision energy of 30, and rapid scan MS analysis in the ion trap. The MS2 ion count target was set to 104 and the max injection time was 35 ms. Precursors with charge state 2-6 were sampled for MS2. The dynamic exclusion duration was set to 45 s with a 10 ppm tolerance around the selected precursor and its isotopes. Monoisotopic precursor selection was turned on. The instrument was run at the top speed mode with 2 s cycles.

All data were analyzed and quantified with the MaxQuant software (version 1.6.3.4). The false discovery rate (FDR) was set to 1% for both proteins and peptides and we specified a minimum peptide length of 7 amino acids. The Andromeda search engine was used for the MS/MS spectra search against the Human database (downloaded from Uniprot on September 2017, containing 20,142 entries). Enzyme specificity was set as C-terminal to Arg and Lys, also allowing cleavage at proline bonds and a maximum of two missed cleavages. Dithiomethylation of cysteine was selected as fixed modification and N- terminal protein acetylation and methionine oxidation as variable modifications. The “match between runs” feature of MaxQuant was used to transfer identifications to other LC-MS/MS runs based on their masses and retention time (maximum deviation 0.7 min) and this was also used in the quantification experiments. Quantifications were performed with the label-free algorithm in MaxQuant.

Data analysis was performed in R after loading the proteinGroups result files from MaxQuant. Proteins with less than 10% valid values were removed. Differentially expressed proteins and their false discovery rate (FDR) corrected p values were identified using the Limma package. We used gene set enrichment analysis (GSEA) as implemented in the clusterProfiler package (version 3.6.0) and gene set variation analysis (GSVA) as implemented in the GSVA R-package (version 1.26.0). Gene set analysis was performed using KEGG gene sets. GSEA and GSVA scores were calculated for sets with a minimum of 5 detected genes, all other parameters were default.

Proteomic raw data are available in the public repository database “open science framework” (https://osf.io/zwx7k/).

### Animal experiments

Aged (18 months) and young (8 weeks) Balb-c or FVB mice (males+females; randomized in groups; purchased from Charles River Laboratories) were treated either with MitoTam (0.5 or 2 mg/kg of body weight), MitoSen (0.5 or 2 mg/kg of body weight) or MitoLab73 (0.5 or 2 mg/kg of body weight), dissolved in 4 % ethanol in corn oil or the vehicle given intraperitoneally (i.p.) once a week for the period of 4 weeks. At the end of the experiment, organs were collected and frozen at -80 °C until further analysis.

All mice were maintained at 22 °C and 12 h/12 h light/dark regimen with food and water provided ad libitum. These experiments were performed in agreement with the Animal Protection Law of the Czech Republic and were approved by the Ethics Committee of the Institute of Molecular Genetics, Prague (permit number 51/2018).

### siRNA- and esiRNA-mediated gene knock-down

Specific siRNAs and esiRNAs were introduced into cells using Lipofectamine™ RNAiMAX (Invitrogen) according to the manufacturer´s protocol. siISCU (s23907, ThermoFisher Scientific) and esiMT-CO1 (EHU117481), esiMT-CO2 (EHU117681), esiMT-CO3 (EHU101051), esiNDUFV1 (EHU033501), esiPLCB4 (EHU087781) and esiLPCAT3 (EHU001051), all from Sigma, were used. siRNA universal negative control (siNC; SIC001; Sigma) was used as a control.

### Statistical analysis

Statistical analysis was performed to assess differences between treatment groups using non-parametric tests. A Kruskal-Wallis test was initially conducted to evaluate overall differences in group medians. Following a significant Kruskal-Wallis result, the Conover-Iman test was used for post-hoc pairwise comparisons between groups. P-values were adjusted for multiple comparisons using the Bonferroni correction method. All statistical calculations were conducted using R version 4.3.0 (R Core Team, 2023. R: A Language and Environment for Statistical Computing. R Foundation for Statistical Computing, Vienna.), with the PMCMR plus package (Pohlert T, 2024. PMCMR plus: Calculate Pairwise Multiple Comparisons of Mean Rank Sums Extended. R package version 1.9.12) for the Conover-Iman test and the stats package for the Kruskal-Wallis test.

## RESULTS

### Triphenyl and tricyclohexyl phosphine modifications of tamoxifen selectively eliminate senescent cells.

To understand how mitochondrial targeting promotes reduction of senescence, we tested various analogs of MitoTam with different phosphine structures to reveal the impact of phosphine structural changes on their biological activity. These compounds consist of both linear (MitoLab57, 72, 73) and cyclic alkyl chains (MitoSen), as well as different aromatic groups (MitoLab54, MitoTam), and their combinations (MitoLab55, 56) (Fig. 1A and Supplementary Fig. 1A). Out of all compounds tested, only MitoSen with tricyclohexyl phosphine as the targeting vector demonstrated a senolytic effect similar to MitoTam containing triphenylphosphine (Fig. 1B, Supplementary Fig. 1B). Their senolytic effect was confirmed to be independent of the cell type or stress that induced cellular senescence (Supplementary Fig. 1C; for the effect of MitoTam on the replicative senescence or radiation induced senescence in fibroblasts see also [3]). This indicates that the structural motif and steric conformation are critical for the senolytic effect favoring triphenyl and tricyclohexyl phosphine above other tested phosphines in combination with tamoxifen since the other bound molecules (e.g. vitamin E succinate) did not show the senolytic effect, as we published prevoiusly [3].

### Mitochondrially targeted senolytic agents affect mitochondrial function in senescent cells.

To demonstrate the senolytic effect of MitoSen *in vivo*, naturally aged (18 month) Balb-c mice (Supplementary Fig. 2A) or FVB mice (Supplementary Fig. 2B) were treated with MitoSen (2mg/kg) once a week for 4 weeks, following the regimen used earlier for MitoTam [3]. In both experimental models, we observed a decrease in senescence levels in the liver and kidneys, which showed the highest accummulation of MitoTam [6], as opposed to MitoLab73, which showed no effect on the senescence level (Supplementary Fig. 2C). We determined comparable effect of MitoTam and MitoSen on senescence reduction also after their reduced dose (Fig. 1C).

In line with previous findings concerning cancer cells [6], both MitoTam and MitoSen inhibited basal (Supplementary Fig. 3A), as well as complex I- (CI) and complex II- (CII) dependent respiration, and complex IV (CIV) activity in senescent RPE-1 cells (Fig. 1D). This was accompanied by decreased mitochondrial potential (Fig. 1E) and changes in mitochondrial morphology observed by confocal (Fig. 1F, Supplementary Fig. 3B) and electron microscopy (Fig. 1G) 48 h after the treatment. These findings correlate with the previously observed ability of MitoTam to affect the inner mitochondrial membrane [7]. However, despite the primary stress, control cells were resistant to cell death and recovered mitochondrial morphology 7 days after treatment with the agents (Figure 1F and Figure S3B), which correlates with our previous observation that MitoTam eliminates senescent cells without permanent effect on cell cycle arrest of control cells [3]. Importantly, while MitoTam and MitoSen reduced respiration in both control and senescent cells, the activity of CIV was unaffected in control cells (Fig. 1D), underscoring the importance of CIV in maintaining mitochondrial integrity, ROS production, and cell survival [8]. Proteomic analysis revealed a decrease in subunits of mitochondrial respiratory complexes I-IV as well as mitochondrial ribosome subunits in senescent cells treated with MitoTam or MitoSen compared to untreated senescent cells (Table 1). These results indicate a complex effect of these compounds on mitochondrial function beyond inhibition of OXPHOS.

**Figure 2.**
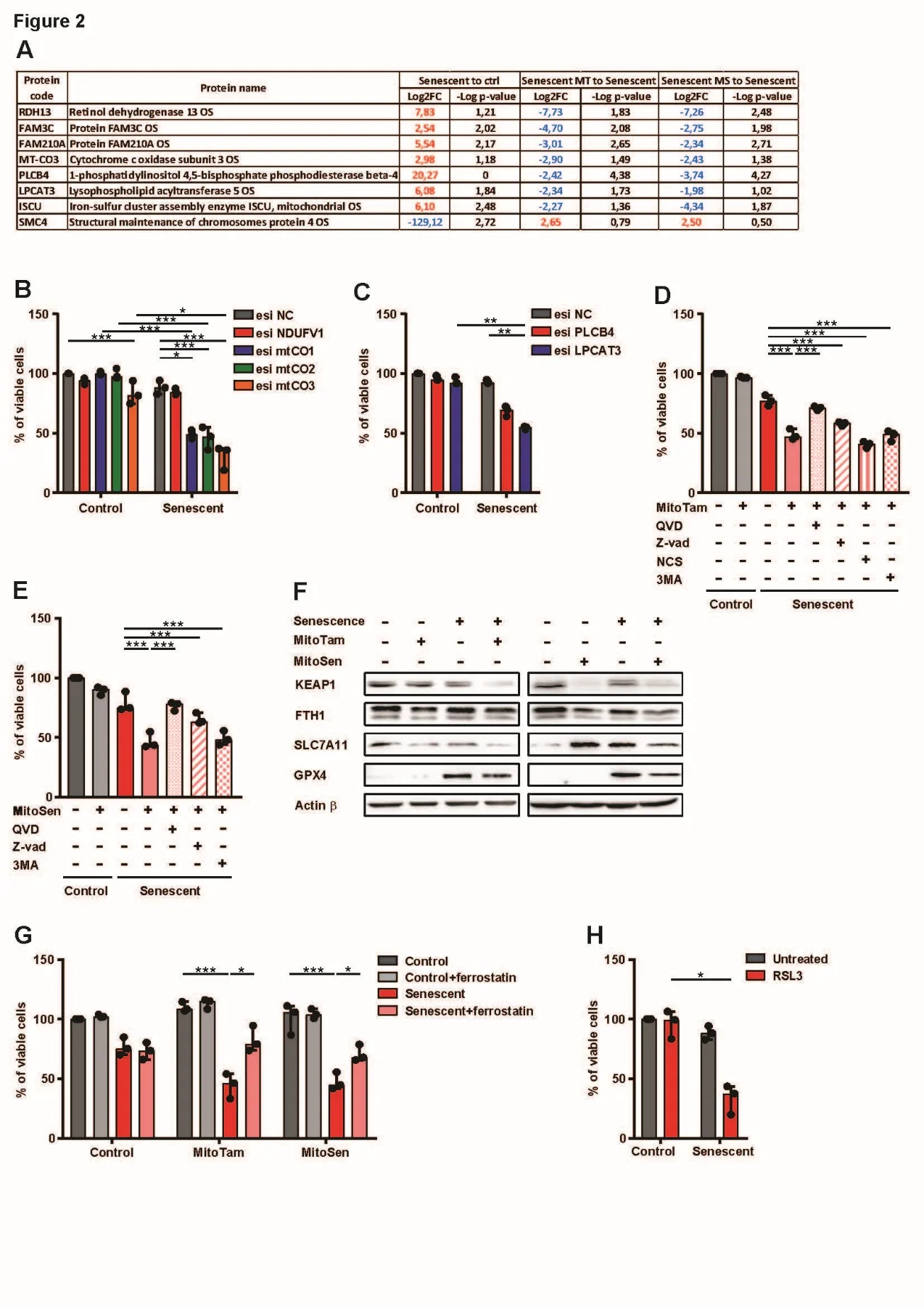
**Mitochondrially targeted senolytic agents induce ferroptosis in senescent cells**. (**A**) Proteomic analysis of RPE-1 control and senescent cells (induced by 100 μM BrdU every 48h for 8 days) treated with MitoTam, MitoSen or MitoLab73 (1μM for all compounds) for 24h. A list of proteins specifically altered in senescent cells compared to control cells and restored in senescent cells following MitoTam and MitoSen treatment is shown. Proteins that exhibited a similar response to the MitoTam or MitoSen treatment in both control and senescent cells were excluded. Proteins that exhibited a similar response to MitoTam or MitoSen and to MitoLab73, which do not affect the viability of senescent cells, were excluded. RPE-1 control, and senescent cells were transfected with specific esiRNAs targeting mRNA of (B) selected mitochondrial respiratory subunits or (C) PLCB4 and LPCAT3 mRNA. Cell viability was assessed 48h post-transfection by WST-1 assay. Universal non-targeting siRNA (siNC) was used as a control. RPE-1 control and senescent cells were pretreated for 30 minutes with pan-caspase inhibitors Q-VD-Oph (50 μM) and Z-VAD-FMK (10 μM), necroptosis inhibitor necrostatin (NCS; 50μM) or autophagy inhibitor 3-methyladenine (3-MA; 1mM), followed by treatment with (D) MitoTam (1μM) or (E) MitoSen (1μM) for 48h. Cell viability was assessed using WST-1 assay. (**F**) RPE-1 control, and senescent cells were treated with MitoTam (1 μM) or MitoSen (1 μM) for 24h. Levels of Kellch-like ECH-associated protein 1 (KEAP1), ferritin heavy chain (FTH1), the cysteine transporter solute carrier family 7 member 11 (SLC7A11), and glutathione peroxidase 4 (GPX4) were analyzed by immunoblot. Actin-β was used as a loading control. (**G**) RPE-1 control, and senescent cells were pretreated for 30 minutes with the ferroptosis inhibitor ferrostatin-1 (1μM), followed by treatment with MitoTam (1μM) or MitoSen (1μM) for 48h. Cell viability was assessed using WST-1 assay. (**H**) RPE-1 control, and senescent cells were treated with the GPX4 inhibitor RSL3 (1μM) for 48h. Cell viability was assessed using WST-1 assay. Data in B, C, D, E, G and H are presented as median values with interquartile range from three independent experiments.

For further analysis, we selected a group of proteins whose expression was altered in senescent cells and was reversed by treatment with MitoTam and MitoSen, but not MitoLab73 (Fig. 2A). Selective downregulation of cytochrome c oxidase 1, 2, or 3 (MT-CO1, MT-CO2, MT-CO3), the catalytic subunits of CIV, specifically decreased viability of senescent cells with only mild effect on control cells (Fig. 2B, Supplementary Fig. 4A). Interestingly, inhibition of NADH dehydrogenase [ubiquinone] flavoprotein 1 (NDUFV1), the catalytic subunit of CI, did not reduce the viability of senescent cells (Fig. 2B, Supplementary Figure 4A), highlighting a unique role of functional CIV in the survival of senescent cells. Moreover, inhibition of 1-phosphatidylinositol-4,5-bisphosphate phosphodiesterase β-4 (PLCB4) and lysophosphatidylcholine acyltransferase 3 (LPCAT3), key regulators of cellular lipid metabolism and mitochondrial membrane phospholipid remodeling and integrity [9], resulted in decreased viability of senescent cells (Fig. 2C, Supplementary Figure 4B). Finally, the lower expression of the iron-sulfur cluster assembly enzyme (ISCU), responsible for iron homeostasis, did not affect the viability of senescent cells (Supplementary Fig. S4C, D).

### Mitochondrially targeted senolytic agents induce ferroptosis in senescent cells.

Similarly as for cancer cells, we detected lack of DNA damage after treatment with MitoTam and MitoSen (Supplementary Fig. 4E). We found that pan-caspase inhibitors block MitoTam and MitoSen-induced death of senescent cells, while inhibition of necroptosis or autophagy had no effect (Fig. 2D, E). As recently described, changes in mitochondrial structure and function are closely linked with ferroptosis [10], a form of caspase-dependent, programmed, non-apoptotic cell death. In senescent cells, ferroptosis is predominantly blocked by the phospholipid peroxidase GPX4 [11], which prevents the accumulation of peroxidized lipids. In our model, both MitoTam and MitoSen not only decreased the levels of GPX4, but also reduced the levels of KEAP1, FTH1, and SLC7A11, other key regulators of ferroptosis (Fig. 2F). Using ferrostatin-1, a specific inhibitor of ferroptosis that mimicks the function of GPX4, we effectively prevented the elimination of senescent cells by MitoTam and MitoSen (Fig. 2G). Conversely, inhibition of GPX4 with the RSL3 inhibitor specifically decreased the viability of senescent, but not control cells (Fig. 2H). Collectively, these data indicate regulation of ferroptosis by MitoTam and MitoSen.

## DISCUSSION

Mitochondria play a pivotal role in both the initiation and maintenance of cellular senescence independently of the type of cell or tissue. Mitochondrial dysfunction, characterized by increased ROS production, altered metabolism, and impaired oxidative phosphorylation, drives the senescent phenotype by exacerbating cellular damage and reinforcing the senescence signaling pathways. The feedback loop between mitochondrial dysfunction and senescence contributes to the decline in tissue function and organismal aging.

**Table 1:** **Proteomic analysis of RPE-1 control and senescent cells (induced by 100 μM BrdU every 48h for 8 days) treated with MitoTam, MitoSen or MitoLab73 (1μM for all compounds) for 24h**. The list of proteins specifically altered in senescent cells following MitoTam and MitoSen treatment is shown. Proteins that exhibited a similar response to the MitoTam or MitoSen treatment in both control and senescent cells were excluded. Proteins that exhibited a similar response to MitoTam or MitoSen and to MitoLab73, which does not affect the viability of senescent cells, were excluded.

Protein code	Protein name	2LogFC	
		Senescent MT to Senescent	Senescent MS to Senescent
NDUFV1	NADH dehydrogenase [ubiquinone] flavoprotein 1, mitochondrial OS	-12,08	-4,37
MRPL28	39S ribosomal protein L28, mitochondrial OS	-10,55	-8,34
MKI67	Proliferation marker protein Ki-67 OS	-9,71	-7,10
FADS2	Acyl-CoA 6-desaturase OS	-8,43	-2,08
RDH13	Retinol dehydrogenase 13 OS	-7,73	-7,26
PTCD3	Pentatricopeptide repeat domain-containing protein 3, mitochondrial OS	-6,13	-3,58
NDUFA5	NADH dehydrogenase [ubiquinone] 1 alpha subcomplex subunit 5 OS	-4,99	-2,86
FAM3C	Protein FAM3C OS	-4,70	-2,75
NDUFS3	NADH dehydrogenase [ubiquinone] iron-sulfur protein 3, mitochondrial OS	-4,03	-2,84
NDUFS2	NADH dehydrogenase [ubiquinone] iron-sulfur protein 2, mitochondrial OS	-3,95	-3,11
MRPS35	28S ribosomal protein S35, mitochondrial OS	-3,89	-2,98
NDUFA10	NADH dehydrogenase [ubiquinone] 1 alpha subcomplex subunit 10, mitochondrial OS	-3,85	-3,36
MRPS31	28S ribosomal protein S31, mitochondrial OS	-3,74	-3,22
MRPL47	39S ribosomal protein L47, mitochondrial OS	-3,58	-4,88
NDUFB3	NADH dehydrogenase [ubiquinone] 1 beta subcomplex subunit 3 OS	-3,55	-2,61
MCM5	DNA replication licensing factor MCM5 OS	-3,49	-2,09
MRPL3	39S ribosomal protein L3, mitochondrial OS	-3,37	-2,73
FAM210A	Protein FAM210A OS	-3,01	-2,34
TBRG4	FAST kinase domain-containing protein 4 OS	-2,95	-2,33
MRPS27	28S ribosomal protein S27, mitochondrial OS	-2,93	-2,04
MRPL32	39S ribosomal protein L32, mitochondrial OS	-2,91	-4,60
MT-CO3	Cytochrome c oxidase subunit 3 OS	-2,90	-2,43
MRPL39	39S ribosomal protein L39, mitochondrial OS	-2,89	-3,64
MRPS5	28S ribosomal protein S5, mitochondrial OS	-2,87	-2,35
NDUFA13	NADH dehydrogenase [ubiquinone] 1 alpha subcomplex subunit 13 OS	-2,79	-2,75
LYZ	Lysozyme C OS	-2,73	-2,78
MRPL17	39S ribosomal protein L17, mitochondrial OS	-2,70	-3,98
MRPL19	39S ribosomal protein L19, mitochondrial OS	-2,63	-3,13
MRPL44	39S ribosomal protein L44, mitochondrial OS	-2,58	-3,23
DAP3	28S ribosomal protein S29, mitochondrial OS	-2,57	-2,84
NDUFB8	NADH dehydrogenase [ubiquinone] 1 beta subcomplex subunit 8, mitochondrial OS	-2,56	-2,45
OAT	Ornithine aminotransferase, mitochondrial OS	-2,55	-2,35
NDUFA3	NADH dehydrogenase [ubiquinone] 1 alpha subcomplex subunit 3 OS	-2,48	-2,90
TFB2M	Dimethyladenosine transferase 2, mitochondrial OS	-2,47	-2,19
MRPS23	28S ribosomal protein S23, mitochondrial OS	-2,45	-3,03
CLPX	ATP-dependent Clp protease ATP-binding subunit clpX-like, mitochondrial OS	-2,43	-2,16
PLCB4	1-phosphatidylinositol 4,5-bisphosphate phosphodiesterase beta-4 OS	-2,42	-3,74
MLF2	Myeloid leukemia factor 2 OS	-2,41	-2,31
NDUFS5	NADH dehydrogenase [ubiquinone] iron-sulfur protein 5 OS	-2,40	-3,51
MRPS18A	39S ribosomal protein S18a, mitochondrial OS	-2,35	-3,53
LPCAT3	Lysophospholipid acyltransferase 5 OS	-2,34	-1,98
NFS1	Cysteine desulfurase, mitochondrial OS	-2,30	-2,08
ISCU	Iron-sulfur cluster assembly enzyme ISCU, mitochondrial OS	-2,27	-4,34
GTF2I	General transcription factor II-I OS	-2,26	-2,10
RBFA	Putative ribosome-binding factor A, mitochondrial OS	-2,15	-3,47
MET	Hepatocyte growth factor receptor OS	-2,14	-2,40
SMU1	WD40 repeat-containing protein SMU1 OS	-2,10	-1,99
MRPL22	39S ribosomal protein L22, mitochondrial OS	-2,09	-2,97
MT-CO1	Cytochrome c oxidase subunit 1 OS	-2,16	-1,57
MT-CO2	Cytochrome c oxidase subunit 2 OS	-1,37	-1,80
SMC4	Structural maintenance of chromosomes protein 4 OS	2,65	2,50
RRM2	Ribonucleoside-diphosphate reductase subunit M2 OS	3,12	3,40
CEBPB	CCAAT/enhancer-binding protein beta OS	3,31	1,96
ANXA4	Annexin A4 OS	3,34	2,01
TNFRSF10B	Tumor necrosis factor receptor superfamily member 10B OS	4,26	2,34
RND3	Rho-related GTP-binding protein RhoE OS	4,89	1,98
CHCHD2	Coiled-coil-helix-coiled-coil-helix domain-containing protein 2 OS	5,45	2,72

Recent research has highlighted the potential of targeting mitochondria as a senolytic strategy. One promising avenue involves the use of small molecules or peptides that can restore mitochondrial function or selectively induce apoptosis in senescent cells by disrupting their mitochondrial integrity. For instance, drugs like inhibitors of Bcl-2 family proteins that regulate mitochondrial membrane stabilization have shown efficacy in killing senescent cells [12]. Additionally, mitochondria-targeted antioxidants are explored to reduce ROS levels and mitigate mitochondrial dysfunction in senescent cells [13]. Targeting mitochondria in senescent cells represents a promising strategy not only to remove these dysfunctional cells but also to improve overall mitochondrial health, thereby enhancing cellular and organismal longevity. Our findings demonstrate that MitoTam and its derivate MitoSen specifically target senescent cells and effectively eliminate them by means of a complex mechanism including reduced mitochondrial function/integrity and activation of ferroptosis. As published previously, the susceptibility of senescent cells to mitochondrially targeted agents is linked to a low expression level of adenine nucleotide translocator-2 (ANT2), a translocator of ADP and ATP across the mitochondrial inner membrane [3]. Unlike ANT1, ANT2 can transport ATP formed by glycolysis into mitochondria to generate the inner membrane potential and maintain mitochondrial function in the state of reduced OXPHOS to evade cell death. The selectivity of our senolytic agents is therefore based on the ability of control cells to maintain mitochondrial integrity via ANT2 in the situation when the mitochondrial network is challenged with MitoTam or MitoSen.

Senescent cells with damaged mitochondria exhibit increased production of hydroperoxides due to elevated levels of reactive oxygen species. Thus, senescent cells have increased levels of antioxidant defense, especially glutathione peroxidase 4 (GPX4), an enzyme that reduces lipid hydroperoxides to non-toxic lipid alcohols, preventing ferroptosis. Our results show that MitoTam and MitoSen decrease the level of GPX4, which contributes to the activation of ferroptosis due to reduced ability of cells to deal with oxidative stress. Moreover, MitoTam and MitoSen reduce the level of KEAP1, FTH1 and SLC7A11, crucial inhibitors of ferroptosis via their role in regulation of GPX4 activity and maintaining redox balance. Although we observed their non-specific downregulation in control and senescent cells, the absence of oxidative stress in control cells prevents these cells from activation of ferroptosis in contrast to senescent cells. This presents a new opportunity for the development of innovative strategies to modulate ferroptosis in senescent cells and mitigate the deleterious effects of senescence on human health. Moreover, accumulation of senescent cells during progression of age-related diseases significantly contributes to cancer development due to their impact on the cellular microenvironment, systemic physiology, and immune function. Conditions like cardiovascular disease, diabetes, and chronic kidney disease are often accompanied by chronic inflammation, oxidative stress, and impaired cellular repair mechanisms, creating a pro-carcinogenic environment. Elimination of senescent cells by MitoTam or MitoSen can therefore not only decrease progression of age-related diseases, as shown in our previous study on diabetes and associated complications [4], but also prevent tumorigenesis.

MitoTam has been originally developed as a new potential anti-cancer drug exploiting the dependence of cancer cells on functional mitochondria for their survival. In 2022 MitoTam has successfully completed the Phase 1/1b MitoTam clinical trial for metastatic solid tumor patients [14]. During Phase 1b of the trial, we observed mostly hematological adverse effects (anemia, neutropenia, thrombocytopenia) as a consequence of the central vein administration, which did not exceed grade 2 and were reversible after the end of the treatment cycle. Preclinical data show MitoTam accumulation in organs such as the kidney, myocardium, lungs, and liver [6]. The clinical study did not show significant organ toxicity, suggesting that MitoTam is generally well-tolerated and has the potential to treat non-malignant diseases. Long-term use of MitoTam for non-malignant indications, however, depends on the ability of its oral administration, which is the subject of intensive research. Oral availability, among other things, will lead to a reduction of its effect on haematopoietic cells.

In conclusion, due to its dual role as both a senolytic and oncolytic agent, MitoTam offers a unique and multifaceted strategy for reducing age-related disorders and preventing/reducing the development of tumors, which are often associated with these conditions.

## Supplementary Materials

The Supplementary data can be found online at: www.aginganddisease.org/EN/10.14336/AD.2024.1100.
