# Supplementary material for: Targeting Mitochondrial Integrity as a New Senolytic Strategy
Source: Aging Dis. 2024 Dec 18;16(6):3638–48. doi: 10.14336/AD.2024.1100 (PMC12539531; doi:10.14336/AD.2024.1100)
Supplement: Supplementary file 1 — The Supplementary data can be found online at: www.aginganddisease.org/EN/10.14336/AD.2024.1100. [file AD-16-6-3638-s.pdf]

SUPPLEMENTARY DATA

Targeting Mitochondrial Integrity as a New Senolytic Strategy

Eliska Vacurova, Edita Vlachova, Jan Stursa, Klara Bohacova, Tereza Havrlantova, Vojtech Skop, Barbora Judita Kasperova, Lukas Werner, Jiri Neuzil, Martin Haluzik, Sona Stemberkova Hubackova

© 2024. Vacurova E. et al. Published online at http://www.aginganddisease.org/EN/10.14336/AD.2024.1100

SUPPLEMENTARY DATA

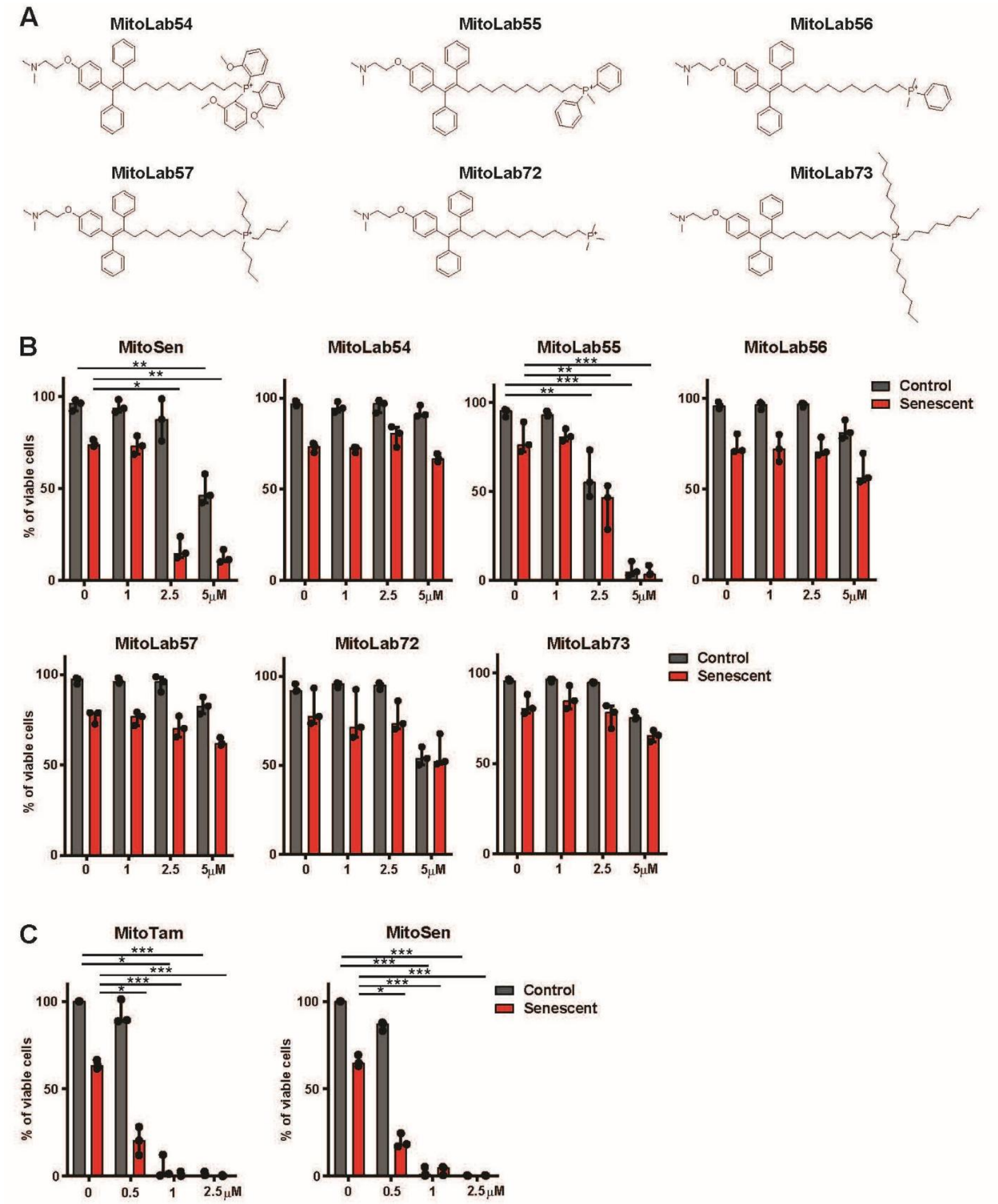

Supplementary Figure 1. (A) Chemical structures of MitoTam derivates. **(B)** RPE-1 control and senescent cells (induced by 100 μM BrdU every 48h for 8 days) were exposed to MitoTam derivates for 48 h at the concentrations as indicated and their viability was evaluated by annexin V/Hoechst negativity using flow cytometry. (C) EA.hy926 control and senescent cells (induced by 100 μM BrdU every 48h for 8 days) were exposed to MitoTam or MitoSen for 48 h at the concentrations as indicated and their viability was evaluated by WST-1 assay. Data in B and C are expressed as median values from three independent experiments with interquartile range.

© 2024. Vacurova E. et al. Published online at http://www.aginganddisease.org/EN/10.14336/AD.2024.1100

SUPPLEMENTARY DATA

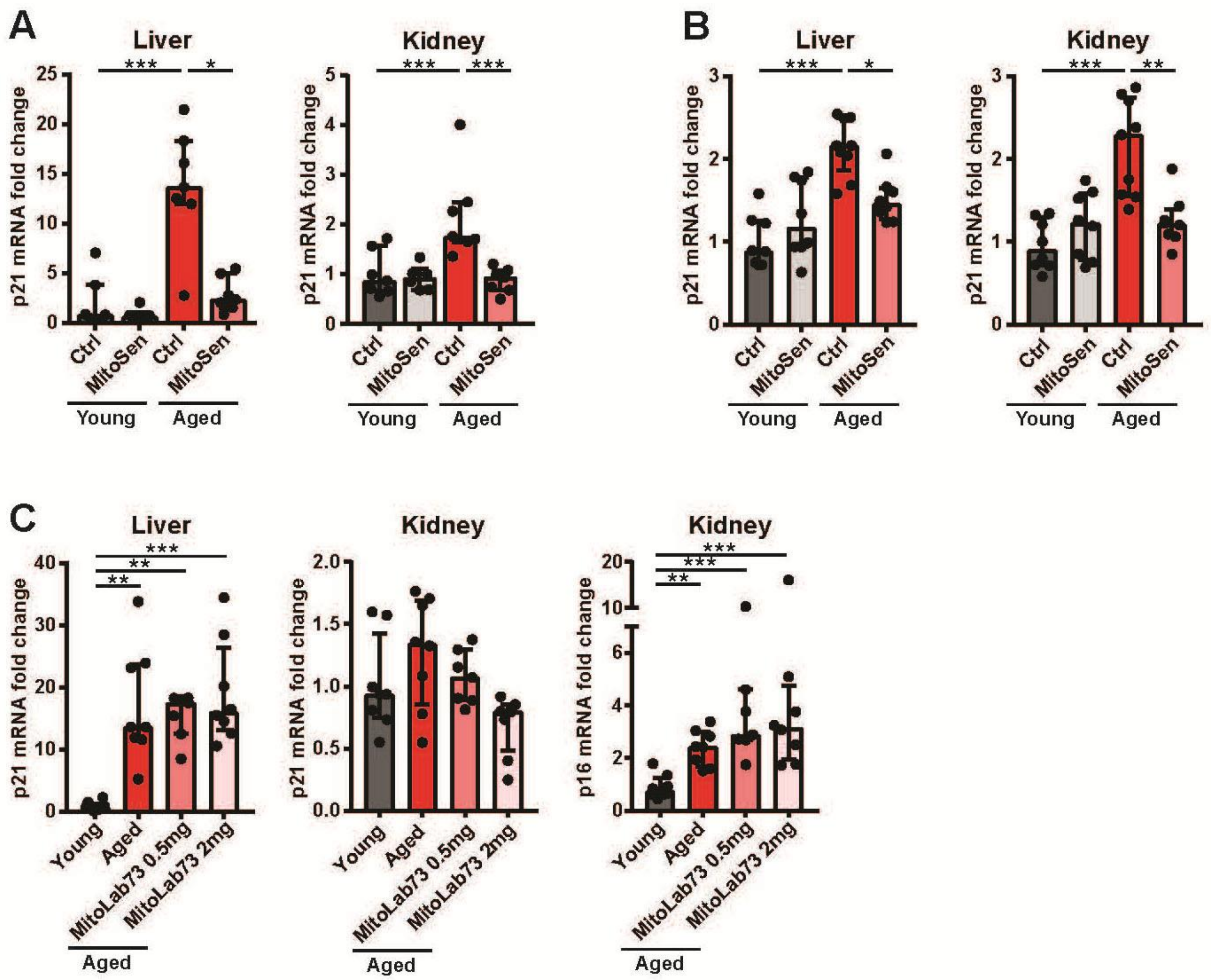

Supplementary Figure 2. (A) Young (8 weeks; n=7) and aged (18 month; n=7) Balb-c mice were given *i.p.* dose of MitoSen (2mg/kg of body weight; n=6 and 7, respectively) dissolved in 4% ethanol in corn oil or the vehicle once per week for four weeks. Expression of $p21^{cip1/waf1}$ in liver and kidney was estimated by RT-qPCR. **(B)** Young (8 weeks; n=8) and aged (18 month; n=9) FVB/N mice were given *i.p.* dose of MitoSen (2mg/kg of body weight; n=8) dissolved in 4% ethanol in corn oil or the vehicle once per week for four weeks. Expression of $p21^{cip1/waf1}$ in liver and kidney was estimated by RT-qPCR. **(C)** Young (8 weeks; n=8) and aged (18 month; n=8) Balb-c mice were given *i.p.* dose of MitoLab73 (0.5 and 2mg/kg of body weight; n=7 and 8, respectively) dissolved in 4% ethanol in corn oil or the vehicle once per week for four weeks. Expression of $p16^{ink4}$ and $p21^{cip1/waf1}$ in liver and kidney was estimated by RT-qPCR. All data are expressed as median values with interquartile range.

© 2024. Vacurova E. et al. Published online at http://www.aginganddisease.org/EN/10.14336/AD.2024.1100

SUPPLEMENTARY DATA

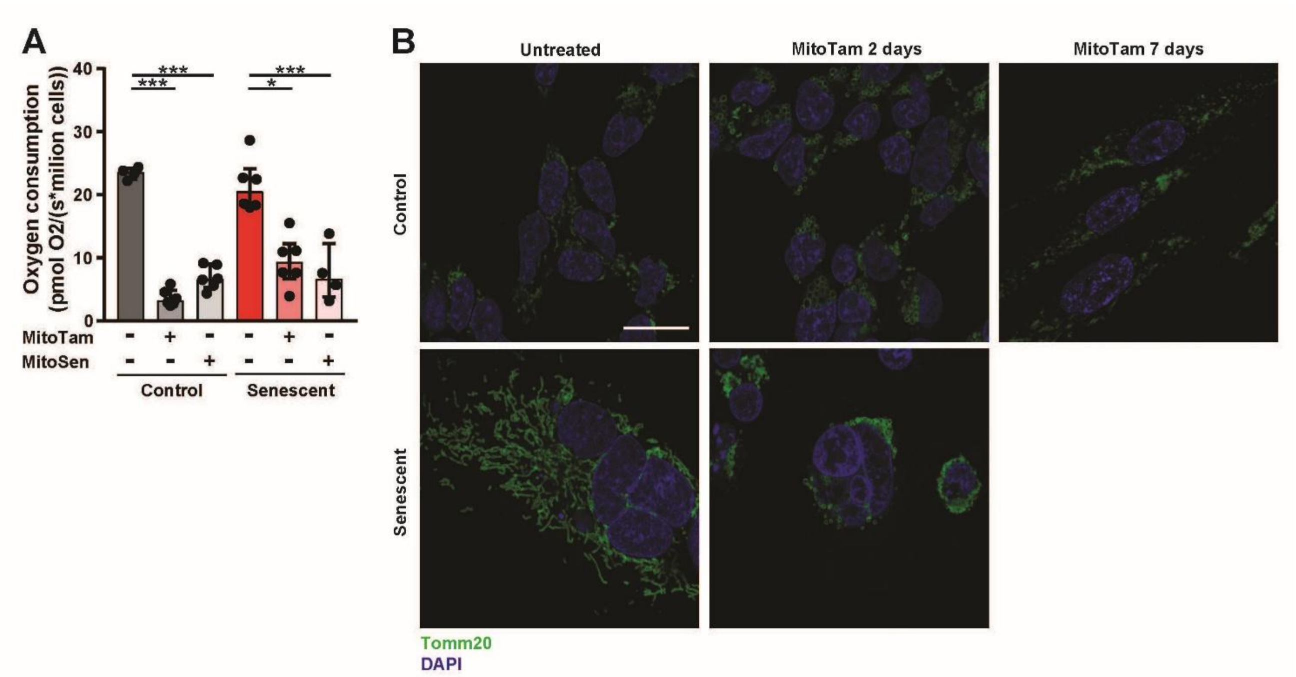

Supplementary Figure 3. RPE-1 control and senescent cells (induced by 100 μM BrdU every 48h for 8 days) were exposed to MitoTam (1 μM) and MitoSen (1 μM) for 24 h. **(A)** Routine respiration was assessed using the Oxygraph 2k instrument. (B) Mitochondrial morphology in RPE-1 control and senescent cells exposed to MitoTam (1 μM) for 24 h or 7 days was documented by Tomm20 immunofluorescent staining, DAPI denoting cell nuclei. The bar indicates 20 μm. Data in A are median values from four to six independent experiments with interquartile range.

© 2024. Vacurova E. et al. Published online at http://www.aginganddisease.org/EN/10.14336/AD.2024.1100

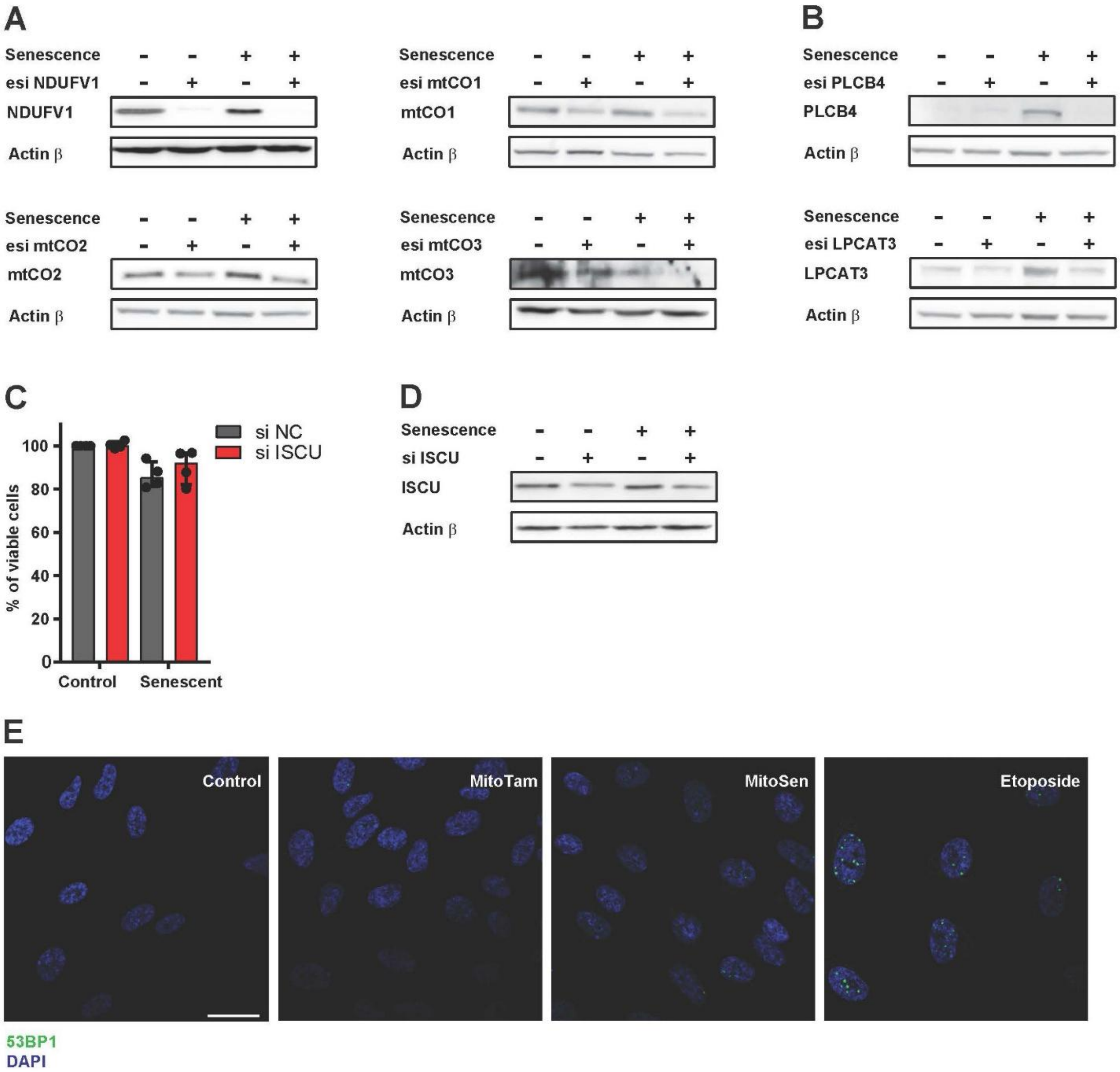

Supplementary Figure 4. RPE-1 control and senescent cells (induced by 100 µM BrdU every 48h for 8 days) were transfected for 48h with esiRNA targeting specific mRNAs as described. **(A)** Levels of NADH dehydrogenase [ubiquinone] flavoprotein 1 (NDUFV1) and cytochrome c oxidase 1, 2 or 3 (MT-CO1, MT-CO2, MT-CO3) or **(B)** 1-Phosphatidylinositol-4,5-bisphosphate phosphodiesterase beta-4 (PLCB4) and Lysophosphatidylcholine acyltransferase 3 (LPCAT3) were analyzed by immunoblot. Actin β was used as a loading control. **(C)** RPE-1 control and senescent cells were transfected with specific siRNAs targeting Iron-sulfur cluster assembly enzyme (ISCU). The percentage of viable cells was assessed 48h post-transfection by WST-1 assay. **(D)** Level of ISCU protein was analyzed by immunoblot. Actin β was used as a loading control. **(E)** RPE-1 control cells were exposed to MitoTam (1 µM), MitoSen (1 µM) and etoposide (5 µM) for 24 h. DNA damage was documented by 53BP1 immunofluorescent staining, DAPI denoting cell nuclei. The bar indicates 20 µm. All data represent three independent experiments. Data in C are expressed as median values with interquartile range.

© 2024. Vacurova E. et al. Published online at http://www.aginganddisease.org/EN/10.14336/AD.2024.1100
